# Prevalence and determinants of self referrals to a District-Regional Hospital in KwaZulu Natal, South Africa: a cross sectional study

**DOI:** 10.11604/pamj.2019.33.4.16963

**Published:** 2019-05-06

**Authors:** Ishandree Pillay, Ozayr Haroon Mahomed

**Affiliations:** 1 Stanger Hospital and Discipline of Public Health Medicine, University of KwaZulu Natal, Durban, South Africa; 2 Discipline of Public Health Medicine; University of KwaZulu Natal, Durban, South Africa

**Keywords:** Self referrals, institutional factors, primary health care

## Abstract

**Introduction:**

Self-referrals to inappropriate levels of care result in an increased patient waiting time, overburdening of higher levels of care, reduced primary healthcare utilisation rate and increasing healthcare costs. Furthermore, self-referral places an additional encumbrance on various levels of care as allocation of resources and infrastructure cannot be accurately planned, based on the facility catchment population. The aim of this study was to determine the prevalence and determinants of patient self-referral at the out-patient department of Stanger Hospital, KwaZulu-Natal between January and June 2017.

**Methods:**

A cross-sectional study was conducted at the out-patient department in Stanger Hospital, using interviewer administered questionnaires to collect information from 385 patients, through convenience sampling, between January and June 2017. Multivariable regression analysis was used to test for factors associated with self-referral.

**Results:**

of the 385 patients interviewed 36% (n = 138) were self-referrals. Most of the self-referrals were male (51.5%) and of the African race (57.2%). Five institutional factors namely: care received from healthcare workers (91.3%); waiting times (88.4%); help offered (87%); treatment and attitude of healthcare workers (63%) and availability of medication (55.8%) were considered as the main drivers of self-referral. Multivariable regression analysis established a significant positive association between patient self-referral and age (40 years and below), attitude of healthcare workers, quality of care received form healthcare workers, waiting times and the availability of diagnostic tests.

**Conclusion:**

This study indicates that most patients attending Stanger Hospital do comply with the prescribed referral pathway, however a significant proportion still bypass the referral system.

## Introduction

**Figure 1:**
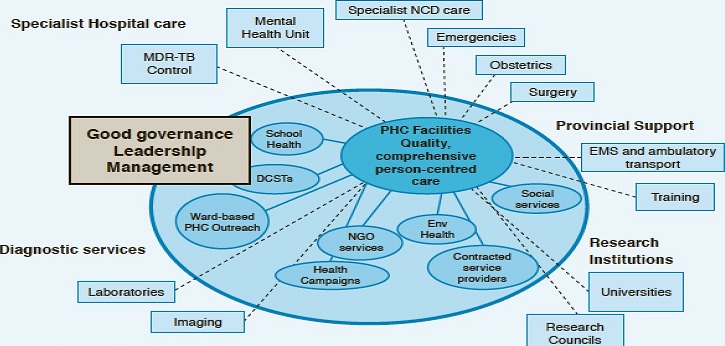
District health system in South Africa (Source: National Department of Health); key: DCST district-based clinical specialist teams; EMS emergency medical services; ENV environmental; MDR-TB multi-drug resistant tuberculosis; NCD non-communicable disease; NGO non-govermental organisation; PHC primary health care

Guided by the Sustainable Development Goals (SDG) [1], Goal 3 and the National Development Plan (NDP) 2030 [2], the National Department of Health (NDoH) has commenced with the implementation of the National Health Insurance (NHI) as a mechanism to achieve Universal Health Coverage (UHC) [3]. Universal health coverage (UHC) means that all people and communities can use the promotive, preventive, curative, rehabilitative and palliative health services they need, of sufficient quality to be effective, while also ensuring that the use of these services does not expose the user to financial hardship [4]. A well-functioning referral system that allows for continuity of care across different tiers of care is central to the delivery of efficient and effective health care and achieving universal health coverage. The Public healthcare system in South Africa is organised in a hierarchical manner with the district health system (DHS) based on the principles of primary health care (PHC) [5] forming the base of the pyramid. Ward Based Outreach PHC teams and Integrated School Health Teams offer health education, health promotion and screening at a community level, whilst the District Clinical Specialist team provide mentoring, supervision, clinical supportive and outreach services for PHC clinics [6]. The PHC clinic is the first formal point of contact with the health services. Patients will be able to present at the PHC clinics with any health care requirement (whether for promotive, preventive, curative; rehabilitative, palliative or community-based mental health) and will either receive the care they need based on the defined package of services at this level or will be referred to a hospital if more specialised services are necessary (Figure 1).

District hospitals form part of the district health system, support primary health care on one hand, and act as a gateway to more specialist care on the other. The district hospital provides level 1 (generalist) services to in-patients and outpatients (ideally on referral from a community health center or clinic) [7]. In some circumstances primary health care services are rendered at the district hospital where there is no alternative source of this care within a reasonable distance [7]. A hierarchical referral relationship between the various levels of hospitals with patients being referred from district hospitals to regional hospitals then to Provincial tertiary hospitals and if required to National Referral Hospitals and Central Hospitals. Specialist, Regional (Level 2), Tertiary (Level 3) and Central (level 4) hospitals provide specialist and sub-specialist services in terms of a defined package of service. A referral system is a comprehensive health care system used to manage client health care needs by referring clients from an initiating facility to an organization, service, or community unit that can better provide the level of care needed. For a referral system to work at its best, all levels of the healthcare delivery system need to be functioning appropriately. Each facility needs to be clear about its role, responsibilities and limitations; have protocols of care for conditions that are specific for that level of service readily available and have suitable means of communication and transport to access support from other levels of care. In 2008, an assessment of the strategic challenges experienced within the South African public health sector revealed numerous systemic problems and concluded that patients were accessing the health system at inappropriate levels and bypassing PHC clinics to attend higher levels of care for their initial visit. This resulted in patients receiving PHC services at regional and tertiary hospitals thus incurring unnecessary costs to the facility [8]. The Kwa-Dukuza municipality services 77% of the district population, but has a low PHC utilisation rate [9]. As Stanger Hospital is the only regional hospital in the Ilembe district and the only hospital in the Kwa-Dukuza Municipality it experiences an influx of patients with medical problems that would be more appropriately managed by the PHC facilities. In 2015, 30,945 patients were self-referred to the hospital [10]. This increased patient load results in increased waiting times for all patients and an increased workload for the affected categories of staff. In the current era of scarce resources, it is important to understand the proportion of patients that are self-referred or inappropriately referred to Stanger Hospital as well as understand the associations between the various factors that affect the referral process. Understanding the referral system and factors associated with compliance are imperative for effective and efficient healthcare planning.

## Methods

**Study design and setting:** a cross-sectional study was conducted at the out-patient department of Stanger Hospital- a 500-bedded regional and district hospital which offers 25 out-patient services. The hospital serves an estimated population of 600 000 from the district.

**Study population and sampling:** a sample size of 385 was calculated using Yamane's formula: n = N/1+N(e)^2 [11]. Using a precision of 5% at the 95% confidence level [12]. Non-probability convenience sampling was used for selecting patients from any of the outpatient departments at Stanger Hospital between January and June 2017.

**Data collection:** data collectors were stationed near the triage desk in the outpatient department, where patients were recruited. Approximately ten patients were interviewed at different times during the day, between 8 am and 4 pm. An interviewer administered questionnaire, in the patients preferred language (English or isiZulu) was administered. The questionnaire included socio-demographic questions, general questions, knowledge questions regarding the referral system, perception of the severity of the condition and clinic and hospital related questions.

**Data processing and analysis:** data was extracted from the questionnaires and thereafter captured using the EpiInfo7software and exported to STATA 13 which was used during the analysis phase. Descriptive statistics in the form of frequencies and proportions for categorical data and measures of central tendency were used for continuous data. The Pearson chi square test was used to determine associations between factors. The multivariate logistic regression model was used to test for associations between dependent and independent variables under study for p-values less than 0.1 after bivariate analysis.

**Ethics and permissions:** ethical approval to conduct this study was granted by the Biomedical Research Ethics Committee of the University of KwaZulu-Natal, Durban (Protocol reference number: BREC REF: BE424/16). Gatekeeper permission was acquired from Stanger Hospital and the Provincial Health Research and Knowledge Management. Informed Consent was obtained from all participants.

## Results

**Figure 2:**
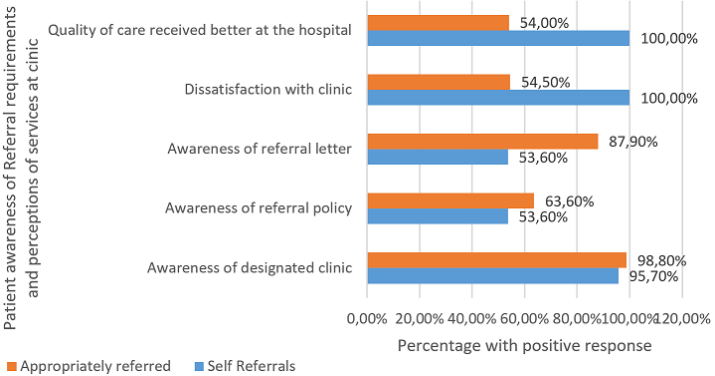
Awareness of referral procedure and perception of quality of care

**Proportion of patients self-referring:** thirty six percent (n = 138) of the patients surveyed self-referred (person who presents at the hospital/ higher level of care for examination, medication or treatment without a referral) to Stanger Hospital. The majority (64%, n = 247) were appropriately referred (Referral for care to an appropriate level in line with the package of services) with all 247 patients producing a referral letter as proof of referral when the researcher requested to see it (Figure 1, Figure 2).

**Socio-demographic profile of study population:** the mean age of the referred and self-referred patients was 44.7 years (SD: 13.3) and 40 years (SD: 14.9) respectively. More males (51.50%, n = 71) self-referred than females (48.50%, n = 67). Majority of the referrals and self-referrals were Black African (67.60%, 57.20%) and Indian (30.00%, 37.00%) respectively. Only two patients (1.50%) in the self-referred group had no formal education however more patients (15.20%, n = 21) in the self-referred group had a tertiary education compared to (7.70%, n = 19) the referred group. One hundred and thirty two patients (53.40%) in the referred group received a salary/ wage in contrast to 43.50% (n = 60) in the self-referred group. Similar to the study population, 68.00% (n = 168) of the patients in the referred group and 66.70% (n=92) in the self-referred group; claim to visit their local clinic first if healthcare is required. A small proportion of patients (1.60%) across both groups reported that they had previously been refused hospital care (Table 1).

**Table 1:** Frequency table of the socio-demographic profile of study population and referred and self-referred patients

Variable	Study population (n n=385)	Referred (n=247)	Self-referred (n=138)
**Age**			
Mean	42.3 (SD: 14.1)	44.7 (SD: 13.3)	40 (SD: 14.9)
Median	41 (IQR: 32-52)	42 (IQR: 35-52)	35 (IQR: 28-54)
**Gender**			
Female	58.70% (226)	64.40% (159)	48.50% (67)
Male	41.30% (159)	35.60% (88)	51.50% (71)
**Race**			
African	64.00%(246)	67.60% (167)	57.20% (79)
Indian	32.40% (125)	30.00% (74)	37.00% (51)
Coloured	2.10% (8)	1.60% (4)	2.90% (4)
White	1.50% (6)	0.80% (2)	2.90% (4)
**Marital Status**			
Single	47.30% (182)	47.80% (118)	46.40% (64)
Married	36.70% (142)	37.30% (92)	36.20% (50)
Divorced	4.00% (15)	2.40% (6)	6.50% (9)
Living With A Partner	12.00% (46)	12.60% (31)	10.90% (15)
**Level Of Education**			
None	0.50% (2)	0% (0)	1.50% (2)
Primary School	12.20% (47)	10.90% (27)	14.50% (20)
High School	76.90% (296)	81.40% (201)	68.80% (95)
Certificate/ Diploma	10.40% (40)	7.70% (19)	15.20% (21)
**Source Of Income**			
None	2.30% (9)	0.40% (1)	5.80% (8)
Salary	44.90% (173)	48.50% (120)	38.40% (53)
Wage	4.90% (19)	4.90% (12)	5.10% (7)
Social Grant/ Pension	18.40% (71)	23.10% (57)	10.10% (14)
Other	29.50% (113)	23.10% (57)	40.60% (56)
**Mode of transport to hospital today**			
Car	21.80% (84)	21.10% (52)	23.20% (32)
Taxi	64.90% (250)	67.60% (167)	60.10% (83)
Bus	6.80% (26)	5.30% (13)	9.40% (13)
Walking	4.20% (16)	2.40% (6)	7.30% (10)
Other	2.30% (9)	3.60% (9)	0% (0)
**Usual Health-Care Provider**			
Phc	16.40% (63)	11.70% (29)	24.60% (34)
Chc	51.20% (197)	56.30% (139)	42.10% (58)
Gp	9.80% (38)	8.50% (21)	12.30% (17)
Local Hospital	22.60% (87)	23.50% (58)	21.00% ( 29)

**Patient awareness of referral policy:** majority of the patients across both the referred (98.80%; 244/247) and self-referred (95.70%; 132/138) groups were aware of their designated clinic, with 157 (63.60%) in the referred and 74 (53.60% in the self-referred groups aware of the hospital referral procedure. More than three quarters of the patients in both groups were aware that a referral letter is required to attend the hospital on the initial visit (Figure 2). One hundred and thirty three (53.80%) of appropriately referred and 100% (138/138) of self-referrals reported that the quality of care in the hospital is better, with 54.50% (135/247) of referrals and 100% (138/138) of self-referrals being dissatisfied with their clinic

**Figure 3:**
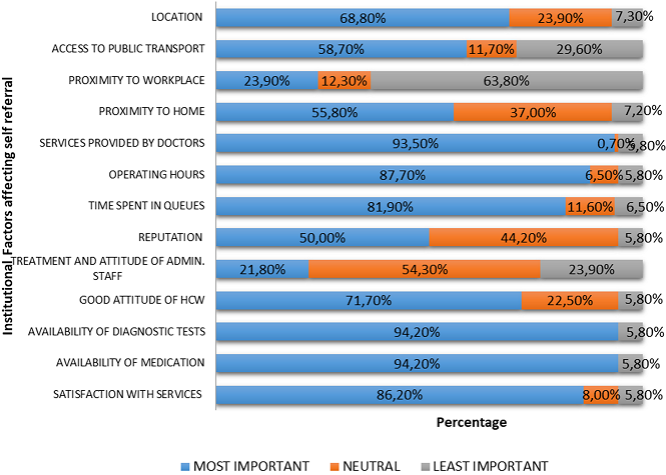
Subjective ranking of institutional factors among self-referred participants

**Subjective ranking of institutional factors among self-referred patients:** the highest ranked institutional factors for self-referrals were: availability of medication (94.20%), availability of diagnostic tests (94.20%), services provided by doctors (93.50%), operating hours (87.70%) and satisfaction with services provided (86.20%) (Figure 3).

**Bi-variate and Multi-variate associations between patient socio-demographic factors and self-referral:** bivariate analysis indicated that Age (40 years and below) (OR 2.31; CI 1.51-3.54, p < 0.001) was significantly associated with self-referral with gender (female), race (African), source of income (permanent employment), awareness of referral procedure and knowledge regarding referral letter were found to be statistically significant (p < 0.1) protective factors. After multivariable analysis; age (40 years and below) (OR 3.29; CI 1.98-5.47, p < 0.001) was significantly associated with self-referral however the gender (female) (OR 0.46; CI 0.29-0.72, p < 0.001), race (African) (OR 0.41; CI 0.25-0.67, p < 0.001), source of income (permanent employment) (OR 0.44; CI 0.26-0.73), p < 0.002) were found to be statistically significant (p < 0.05) protective factors (Table 2).

**Table 2:** Bivariate and multivariable analysis assessing patient socio-demographic factors and self-referral in Stanger Hospital

Variable	Bivariate analysis			Multivariate analysis		
	Un-adjusted OR	P-Value	95% CI	Adjusted OR	P-Value	95% CI
**Age**						
40 years and below	2.31	<0.001*	1.51-3.54	3.29	<0.001**	1.98-5.47
**Gender**						
Female	0.52	0.003*	0.34-0.80	0.46	0.001**	0.29-0.72
**Race**						
African	0.64	0.043*	0.42-0.99	0.41	<0.001**	0.25-0.67
**Marital status**						
Single	0.94	0.792	0.62-1.44			
**Level of education**						
High school and above	0.65	0.159	0.35-1.19			
**Source of income**						
Permanent employment	0.67	0.061*	0.44-1.02	0.44	0.002**	0.26-0.73
**Monthly income**						
Below R3000	0.86	0.472	0.57-1.30			
**Family support**						
Yes	0.64	0.16	0.34-1.20			
**Awareness of referral procedure**						
Yes	0.66	0.057*	0.43-1.01	1.02	0.939	0.58-1.79
**Knowledge regarding referral letter**						
Yes	0.46	0.005*	0.26-0.79	0.54	0.091	0.27-1.10

* = level of statistical significance p<0.1

** = level of statistical significance p<0.05

**Bi-variate and Multi-variate associations between institutional factors and self-referral:** bivariate analysis indicated that waiting times (OR 4.27; CI 2,59-7,04, p < 0.001); availability of diagnostic tests (OR 2,51; CI 1,12-5,59, p = 0.03) and availability of medication (OR: 2,08 CI: 0,92-4,69, p = 0,08) were found to be significantly associated with self-referral. After multivariable analysis only waiting times (OR 6.41; CI 3.42-11.99, p < 0.001) and availability of diagnostic tests (OR 13.22; CI 2.09-83.82, p = 0.006) were found to be significantly associated with self-referral. Reputation of the facility (OR 0.23; CI 0.13-0.39, p < 0.001) and availability of medication (OR 0.12; CI 0.17-0.79, p = 0.028) were found to be protective factors (Table 3).

**Table 3:** Bivariate and Multivariable analysis assessing the hospital factors and self-referral

	Bivariate analysis			Multivariate analysis		
Variable	Unadjusted OR	P-Value	95% CI	Adjusted OR	P-Value	95% CI
Location	1.22	0.375	0.78-1.91			
Access to public transport	0.92	0.70	0.60-1.41			
Proximity to home	1.27	0.259	0.84-1.93			
Proximity to work place	0.88	0.604	0.54-1.43			
Services provided bydoctors	1.69	0.194	0.77-3.71			
Operating hours	1.42	0.262	0.77-2.60			
Waiting times	4.27	<0.001*	2.59-7.04	6.41	<0.001**	3.42-11.99
Reputation	0.47	0.001*	0.31-0.72	0.23	<0.001**	0.13-039
Treatment and attitude of administrative staff	0.56	0.018*	0.34-0.91			
Treatments and attitude of healthcare workers	1.24	0.358	0.79-1.95			
Availability of diagnostic tests	2.51	0.025*	1.12-5.59	13.22	0.006**	2.09-83.82
Availability of medication	2.08	0.079*	0.92-4.69	0.12	0.028**	0.17-0.79
Satisfaction with services	1.17	0.595	0.65-2.12			

* = level of statistical significance p<0.1

** = level of statistical significance p<0.05

## Discussion

The KwaZulu Department of Health has set targets of 7.5% and 6.5% respectively for “new case not referred”; during 2015/16 and 2016/17 in order to improve the operational efficiency of the healthcare delivery system. The findings of the current study (36%) are 3.6 times higher than the current reported rate of 10%, as indicated by the KZN DOH [13]. This is likely attributed to Stanger Hospital being the only regional hospital in the Ilembe district and the only hospital in the Kwa-Dukuza Municipality, which contributes to an influx of patients with medical problems that would be more appropriately managed by the PHC facilities. This finding is consistent with both, a 2009 study conducted at a teaching hospital in Ghana that established a self-referral rate of 33.9% [14] and a 2008 national study conducted in the USA which found that 32% of the respondents bypassed the PHC level [15]. Conversely, the current study findings are significantly lower than that of two Western Cape studies conducted at the G.F. Jooste Hospital and George Provincial Hospital which established a self-referral rate of 80% [16] and 88.9% respectively Patients' failure to recognize the prescribed pathway and its implications on the healthcare system have been highlighted as a significant reason for patients bypassing lower levels of care [17-21]. Multivariable analysis established no significance between self-referral and the awareness of the referral procedure (OR 1.02; CI: 0.58-1.79, p=0.939) and referral letter for hospital attendance (OR 0.54; CI: 0.27-1.10, p = 0.091).

However, a Gauteng based study conducted among maternity patients showed that a large proportion of patients bypassed lower levels of care due to their lack of awareness regarding the referral system [21]. Contrary to this, a 2010 cross-sectional study in KZN established that the 76% of the patients were aware of the existence of a referral system between their local clinic and hospital [22]. This finding suggests that awareness alone may not be adequate to reduce or prevent self-referrals. In the current study 53.80% of the referrals and 100% of the self-referrals reported that the hospital provides a better service than their designated clinic. Similar results were reported among self-referrals to a Western Cape hospital where 23.7% of the patients felt that treatment at the hospital was better [23]. In addition a study investigating referral patterns in Namibia revealed that 36% of patients sought treatment outside of their district for better quality care [24]. As a result, patients may be prepared to spend more time and money to attend a healthcare facility which they perceive offers better care. Such inferences have implications for future interventions in terms of improving patient perception of lower levels of care and improving services at the proximal facilities.

### Patient socio-demographic factors and self-referral

Self-referral is higher among younger participants 40 years and below of age versus participants above 40 years (OR 3.29; CI 1.98-5.47, p < 0.001). These results are corroborated by findings from a 2014 Dutch cohort study that found that 69% of self-referrals were less than 35 years old [25], whilst a descriptive cross-sectional study found that most of their self-referrals (79%) were below the age of 45 [26] and a study conducted in Limpopo where 59% of patients between the ages of 20-39 years had bypassed their local clinic [27]. This may be accredited to older patients experiencing more difficulty travelling or having stronger ties with local professionals [28]. More males (51.5%; n = 71) self-referred than females (48.5; n = 67). This finding is similar to an Ethiopian study [29] and two US studies that reflected that males who are employed tend to bypass the referral system more frequently in comparison to females [30, 31]. In addition, the health seeking behaviour of men is influenced by practical, financial and behavioural reasons [32]. In South Africa, the overwhelming majority of the nurses are women with primary healthcare being a predominantly nurse led service. With deeply socialised stereotypes about gender and health, men's attitudes towards the health sector are further complicated by this uneven representation of men and the skewed distribution and organisation of primary healthcare in favour of women. Therefore men are less likely visit primary health centres in favour of hospitals.

However, these findings are contrary to studies conducted in Brazil and Kenya which showed that a higher percentage of females utilised the A&E department more frequently and inappropriately [33, 34]. In addition, local studies have consistently reported a higher proportion of females who self-refer. In a KZN study conducted in the Umuziwabantu sub-district, 77% of females had self-referred to a district hospital [22] whilst a study based in the Greater Tzaneen municipality showed that 68% of self-referrals were female [27]. The higher proportion of female self-referral may be due to females having an increased health-seeking behaviour when compared to males [35] and possibly because of the perception of quality maternal and newborn health services offered particularly during delivery [36]. Some studies have indicated that the difference in perception of health risk between males and females can contribute to the phenomenon of bypassing [37]. This study did not find any significant association between a patients level of education (high school education and above) (OR 0.65; CI 0.35-1.19, p = 0.159) and source of income (permanent employment) (OR 0.67; CI 0.44-1.02, p = 0.061) to self-referral. The correlation between a patient's level of education and self-referral is inconsistent globally. Studies conducted in higher income countries like the USA and Canada found that patients with higher levels of education were more likely to self-refer [38, 39] however the inverse was established in middle to lower income countries [18, 29]. The findings from a 2015 study, conducted at the Dilokong Hospital, noted that 31.4% of the unemployed patients self-referred as opposed to 24.7% of the employed patients, however this factor was not significant after statistical analysis [40]. Similar findings were observed in other South African studies were over half of the self-referrals were unemployed [22, 27]. In addition, US and Dutch study found a trend between patients from higher socio-economic class and self-referral [41, 42] while other African studies have shown that patients from higher socio-economic groups utilised higher levels of care less frequently [43, 44]. This may be attributed to the advanced resources and better quality services being provided in higher income countries whereas the latter is possibly as a result of these individuals having access to private health care.

### Institutional factors influencing self-referral

**Waiting times**: the time taken to receive medical attention at a facility has been frequently cited to influence healthcare utilisation. In this study, the waiting time at a healthcare facility was found to be highly important among the self-referred patients (88.40%). Similarly, a Kenyan study showed that that patients who were satisfied with their facility waiting times were less likely to bypass that facility [45]. This inference is shared by other South African studies that reported increased waiting times at their local facilities encouraged bypass behaviour [22, 23, 26]. Although waiting time has been established as a key institutional factor; self-referrals further increases the waiting time at higher levels of care. However, majority of the respondents from a KZN study found the self-referred facility waiting time more acceptable in comparison to time spent waiting at their local clinic [22].

**Availability of medication:** in this study the availability of medication at the pharmacy was noted as an important determinant for self-referral in both the referred and self-referred groups (34.40% vs. 55.80%). These findings are consistent with studies in Tanzania and in Kenya; were both showed that dissatisfaction with the shortage of medicines in facilities affects health-seeking behaviour [46, 47]. Contrary to these findings, 41% of patients from a study conducted in Limpopo reported that their local clinic consistently experienced pharmaceutical stock-outs, however only 5.1% of the patients in this study cited this as the reason for self-referral [27]. These findings indicate that patient self-referral may be driven by a perception of lack of medication at local clinics. This false perception increases the likelihood to self-refer, resulting in underutilised PHC services.

**Study limitations:** although due diligence was maintained to ensure the integrity of the study, the findings of the study are influenced by a number of limitations. These included amongst other the reluctance of patients to participate and/or reluctance to disclose all information as they may have felt that it would negatively impact their current/ future healthcare service. In addition the study design provided only a snap shot of the determinants of self-directed referrals among a sample of patients attending Stanger Hospital and the study was conducted over a period of six months.

## Conclusion

The majority (64%) of patients seeking medical attention in the study were adherent to the prescribed referral pathway; while only 36% self-referred during the study period. Multivariable regression analysis established a significant positive association between patient self-referral and age (40 years and below), attitude of healthcare workers, quality of care received form healthcare workers, waiting times and the availability of diagnostic tests. This relationship between the individual and institutional factors suggests interventions at both a health system and an individual level are required to improve compliance to referral procedures. Mobile technology could be used as a social marketing campaign to promote use of the PHC facility. Furthermore the use of an electronic health record will serve as a tool to assure patients of the continuity of care that will be received even if they are consulted at the nearest PHC facility.

### What is known about this topic

- A referral system is a comprehensive health care system used to manage client health care needs by referring clients from an initiating facility to an organization, service, or community unit that can better provide the level of care needed;
- For a referral system to work at its best, all levels of the healthcare delivery system need to be functioning appropriately. Each facility needs to be clear about its role, responsibilities and limitations; have protocols of care for conditions that are specific for that level of service readily available and have suitable means of communication and transport to access support from other levels of care;
- An assessment of the strategic challenges experienced within the South African public health sector revealed numerous systemic problems and concluded that patients were accessing the health system at inappropriate levels and bypassing PHC clinics to attend higher levels of care for their initial visit.

### What this study adds

- Thirty six percent of patients bypass the referral system;
- Significant positive association between patient self-referral and age (40 years and below), attitude of healthcare workers, quality of care received form healthcare workers, waiting times and the availability of diagnostic tests.

## Competing interests

The authors declare no competing interests.
